# ERP evidence of heightened attentional response to visual stimuli in migraine headache disorders

**DOI:** 10.1007/s00221-022-06408-5

**Published:** 2022-08-11

**Authors:** Faly Golshan, Daneil Moss, Gloria Sun, Olav Krigolson, Maria T. Cruz, Janeen Loehr, Marla Mickleborough

**Affiliations:** 1 Department of Psychology and Health Studies, University of Saskatchewan, 105 Administration Place, Saskatoon, SK S7N 5A2 Canada; 2 College of Medicine, University of Saskatchewan, Saskatoon, Canada; 3 University of Victoria, Victoria, Canada

**Keywords:** Migraine, Visual attention, Event-related potentials, Oddball paradigm

## Abstract

New findings from migraine studies have indicated that this common headache disorder is associated with anomalies in attentional processing. In tandem with the previous explorations, this study will provide evidence to show that visual attention is impacted by migraine headache disorders. 43 individuals were initially recruited in the migraine group and 33 people with non-migraine headache disorders were in the control group. The event-related potentials (ERP) of the participants were calculated using data from a visual oddball paradigm task. By analyzing the N200 and P300 ERP components, migraineurs, as compared to controls, had an exaggerated oddball response showing increased amplitude in N200 and P300 difference scores for the oddball vs. standard, while the latencies of the two components remained the same in the migraine and control groups. We then looked at two classifications of migraine with and without aura compared to non-migraine controls. One-Way ANOVA analysis of the two migraine groups and the non-migraine control group showed that the different level of N200 and P300 amplitude mean scores was greater between migraineurs without aura and the control group while these components’ latency remained the same relatively in the three groups. Our results give more neurophysiological support that people with migraine headaches have altered processing of visual attention.

## Introduction

Migraine is a well-known neurological headache disorder that is often characterized by many physiological symptoms including light sensitivity, nausea, and throbbing headaches during headache attacks—also known as ictal periods. Among all different types of this primary headache disorder, migraines with aura cause more pronounced clinical symptoms and have diverse manifestations that vary from visual disturbances to paresthesia or speech disturbances, with visual auras as the most common symptom with 90% of occurrence in this subcategory (Dodick 2018). Migraines without aura are a more recurrent type of migraine with less visual phenomena and include menstrual migraines (Headache Classification Committee of the International Headache Society 2018). Migraines are predominantly reported to cause not only adverse pain but also subjective impairments in cognition (Vuralli et al. 2018). Although the memory and cognitive interruptions are usually found before (pre-ictal) and during (ictal) headache attacks, studies show the cognitive dysfunctions could last between the main attacks (known as the interictal phase) (Mickleborough et al. 2014).

Visual attention is one of the most critical cognitive processes and is frequently found to be impaired based on the subjective clinical reports of migraine sufferers. Attention is the way we focus our cognitive resources on aspects of the visual world that are behaviourally relevant to our current task while diminishing the extent to which we are distracted by less relevant inputs. Research from the past decade has started to paint a picture that while controls readily identify behaviourally relevant stimuli and suppress non-relevant stimuli, migraineurs have a decrease in the ability to suppress behavioral irrelevant stimuli (Mickleborough et al. 2011). Specifically, it is found that the migraine brain shows altered habituation to repeated visual stimuli (Fong et al., 2020; Guo et al., 2019a, b; Coppola et al. 2019). For example, Mickleborough et al. (2013) used logos as visual stimuli for 25 migraineurs and 25 non-migraineurs to compare their post-sensory processing. While viewing the repeatedly presented images, the migraine group had an amplified cortical response to the repeated visual stimuli while the controls showed almost no change in brain response across the same repeated stimuli. Yet the current literature shows contradictory results regarding whether migraine causes an amplification (Mickleborough et al. 2013; Coppola et al. 2009; Chen et al. 2020; Kam et al. 2015) or an attenuation of attentional processing (Guo et al. 2019a, b; Raggi and Ferri 2020), suggesting that more supporting research is needed to show the potential impairment and attentional dysfunction in migraineurs. Recent research has identified a need for continued research assessing altered habituated responses in the evoked potentials of migraineurs vs. non-migraine controls to explore whether the failure for habituated responses could be reproducible in different contexts including with different stimulation parameters (Sand et al. 2008; Omland et al. 2016).

Evidence is building that migraineurs have attentional issues specific to the decision to suppress responses to behaviourally irrelevant stimuli. As an example, Chen et al. (2020) described dysfunctional inhibitory control in migraineurs when suppression of response is expected, and Mickleborough et al (2011) report migraineurs have a significant decrease in normal suppression of cortical responses to visual events outside their zone of attentional. It is not surprising then to find that migraineurs also have increased attentional orienting to sudden-onset stimuli as compared to controls (Mickleborough et al. 2011). Adding to this, previous neuroimaging studies exhibit the altered cortical activities in the attentional control network of migraineurs during visuo-spatial tasks including regions such as the frontal eye field, superior parietal lobule, superior temporal gyrus, and superior temporal sulcus (Kelley et al. 2007; Mickleborough et al. 2016). During visual spatial-orienting tasks in controls as compared to migraineurs, Mickleborough et al. (2016) found that controls have more activation than migraineurs (in between attacks) in the right temporal-parietal junction (rTPJ), a key area in the visual attentional network which has a suggested role of assessing unattended stimuli for behavioural relevance before sending the signal to redirect attention to behaviourally relevant sensory stimuli that are outside the focus of attention (Corbetta et al. 2000; Giesbrecht et al. 2006; Kelley et al. 2007). Therefore, the decreased activity in the rTPJ (in migraineurs as compared to controls) supports the theory that migraineurs have attentional issues related to processing behavioural significance of stimuli and selecting irrelevant stimuli to ignore. Given this growing evidence that the migraine brain may not be adequately assessing the relevance of behavioral stimuli, impacting the amount to which it is attended to, suppressed or habituated to, a *sustained attention paradigm* is an appropriate task to further understand migraineurs dysfunctional attention.

The *Hillyard sustained attention paradigm* is a common experimental design which includes a sequence of auditory or visual repetitive stimuli that are randomly interrupted with a deviant stimulus, such an interruption can cause a measurable change to event-related potential (ERP) components with predictable negative and positive peaks (i.e., amplitude) and timing (also called latency) after the onset of the stimulus (Luck and Kappenman 2017). While the amplitude of each component indicates the intensity of its relevant sensory or cognitive response, the shortened or prolonged duration of a component is shown in its latency which could reflect separate indications about the individual’s response. The visual oddball paradigm task is an example of this experimental design where participants are given multiple visual stimuli (such as letters, shapes, or colours) that could appear on the central fixation point or in other visual fields depending on which specific type of attention (such as visuo-spatial attention, or involuntary attention) is investigated. Right after the onset of the stimulus in an oddball paradigm, the early components with negative and positive polarity are attributed to the motor response and sensory processing of the input, always followed by the emergence of other components that indicate higher cognitive processing as the main focus of attention studies.

The ERP analysis of these cognitive components could help us explore whether migraineurs have an amplified or attenuated excitatory mode in response to the repeated environmental input. ERPs are prominent non-invasive measurements of attention-related brain activities and include components of the continuous EEG recording that occurs immediately after the onset of a stimulus in an attention task. If an individual is shown a series of stimuli, ERP components could be measured by averaging the recurrent EEG activity that occurs after each stimulus, resulting in positive or negative polarities time-locked to the stimulus onset (Luck and Kappenman 2017). ERPs are an appropriate choice for our attention research in migraineurs, given their clear temporal resolution of brain activities (Luck and Kappenman 2017).

The N200 component of ERP is the negative polarity that usually indicates cognitive processing before the motor response to a stimulus as well as object recognition (Woodman 2010) after the onset of a visual stimulus. This early exogenous component is generally associated with involuntary and unwanted processing of the stimuli (Patel and Azzam 2005) but compared to other components such as P300, it is not broadly discussed in the migraine population and needs more investigations as a component of interest especially in the context of visual attention. An early study by Drake et al. (1989) focused on auditory event-related potentials giving 30 unmedicated migraineurs and 20 controls various tones at 1000 and 3000 Hz. They showed no difference in N200 amplitude (the minimal peak) and latency (the time difference between the stimulus onset and the peak amplitude) in migraineurs. On the other hand, Coppola et al. (2019) and de Tommaso et al. (2014) focused on the somatosensory stimulation of migraine without aura and showed that when migraineurs without aura are given a high-frequency repetitive transcranial magnetic stimulation (HF-rTMS), the evoked potential showed a heightened N200 and P250 in high frequency somatosensory evoked potentials of the migraineurs without aura while these components showed a decreasing response to HF-rTMS in healthy controls. On the other hand, Fong et al. (2020) associated the increase in N200 amplitude with cortical hyperexcitability that could happen in some non-migraineurs comparable to migraineurs.

Here in this study, we will explore whether there could be an alteration of the N200 component during a visual oddball task when we compare migraineurs to non-migraine headache sufferers. Reports on N200 latency are mainly controversial indicating shortened, heightened or unchanged 200 timing among migraineurs, such inconsistency could be associated with more sensory processing imbalance caused in visual pathways (Guo et al. 2019a, b; Fong et al. 2020).

The P300 component of ERP data is a key component of interest when assessing late exogenous ERPS and visual attention processes (Krigolson et al. 2017). The P300 is best elicited in oddball paradigms and is especially sensitive to active attentional processing, which makes it a clear variable for examination in a study focused on group differences in visual attention (Krigolson et al. 2017). As is indicated by the name, the P300 ERP is shown graphically as a positive fluctuation as early as 300 ms after the presentation of a stimulus. This component is mostly associated with stimulus encoding, identification and categorization (Picton 1992). Furthermore, the P300 ERP component can determine whether an individual is attending to a stimulus by demonstrating activities near the 300 ms window (Krigolson et al. 2017). For example, when someone is mind wandering, their P300 amplitude is reduced compared to when they are given a task-relevant stimulus (Kam et al. 2015). Regarding this, a look at this component could help us discover how much the participants are involved with attentional processing. Some studies have focused on this component in migraineurs and the findings controversially show increased (Mickleborough et al. 2013) or decreased P300 peak amplitude (Guo et al., 2019b). Both attenuation or amplification of P300 component indicate an altered attentional processing of the migraineurs. In addition to P300 amplitude, the latency of this component is used as an indication of the time individuals would spend on discriminating or categorizing the standard stimulus from the oddball stimulus (Fong et al. 2020). Although Drake et al. (1989) earlier reported that P300 could be prolonged for migraineurs with reference to more expected stimulus processing time in migraineurs, we would like to measure migraineurs and non-migraineurs’ P300 latency in an oddball paradigm.

While recent studies with a focus on attentional processing in the oddball paradigm have compared migraineurs with healthy controls showing alterations in N200 and P300 components in terms of their amplitude and latency (Vilà-Balló et al. 2021; Guo et al. 2019a, b), we further explore if there are any differences between migraine population and other non-migraine headache sufferers.

The current study utilizes a 15-min visual oddball paradigm task presentation along with an electrode headband—the MUSE EEG system—to analyze the frontal and temporal ERP activities of participants. This system has been previously used by Krigolson et al. (2017) for the same oddball task, and we are now applying it to migraine vs. non-migraine groups. Such a portable system could provide more context-based information about attentional processing in different everyday life settings. This study is an opening exploration of a portable EEG headband to collect the fronto-temporal activities of migraineurs during a visual attention task. Given that migraineurs have an altered response to repeated visual stimuli, we hypothesize that they will show amplification of the normal oddball response. We would like to address the current gap of a clear temporal picture about how cognitive processing is altered in the migraineurs and whether such predicted hyperexcitable may differ in different types of headaches comparing migraineurs to non-migraine headache sufferers as well as migraineurs without-aura to migraineurs with aura who usually report more neurological symptoms. Regarding the collected ERP data, we expect the migraine brain will have an even greater increase in attentional response as measured via ERP N200 and P300 components as compared to controls oddball stimuli appear in a series of standard stimuli. Additionally, we will explore how these components differ if comparing two migraine headache categories (with and without aura) with the non-migraine headache controls.

## Methods

### Study design

We chose a cross-sectional study design to observe the attentional response of migraineurs compared to non-migraine headache sufferers in response to a visual oddball paradigm task. We subsequently categorized migraineurs into migraineurs with and without aura to learn how these two groups would show different levels of response compared to the control group. Our dependent variables included N200 and P300 components of the collected ERP while the respondents were given a repetitive visual oddball paradigm task.

### Participants

The current study recruited undergraduate students from the University of Saskatchewan. All the participants were rewarded 1 course credit or a gift card honorarium for their voluntary participation. All the participants gave their written consent form approved by the Biomedical Research Ethics Board at the University of Saskatchewan (Bio-REB 652) before they filled out a self-report questionnaire on their headache characteristics and had a quick tutorial (less than 30 min) on the MUSE headband and their oddball paradigm task. A total of 85 participants were initially recruited for this study all of whom reported some headache experiences. After data collection, five participants were eliminated due to missing or poor EEG data, one who had taken medicines less than 24 h before testing, two participants with headache experience within the past 48 h prior to data collection, and two individuals with probable migraine but not enough symptoms to be considered in the migraine group. Based on the headache criteria of the International Classification of Headache Disorders guide (Headache Classification Committee of the International Headache Society 2018), we analyzed 75 participants (57 females, 15 males and 3 unspecified, age > 18 with a mean age of 13 as the onset of their headache symptoms), who were placed in migraine (*n* = 42) and the non-migraine headache control groups (*n* = 33). 33 identified female (78.5%) and 8 male (19.04%) were in the migraine group and 24 female (72.7%) and 7 male (21.2%) were included in the non-migraine group. The female to male ratios in our groups reflect reports that migraine is reported to be almost three times more prevalent among women than the males (Al-Hassany et al. 2020). The groups were matched as a function of age and education.

### Reported headache characteristics

The participants initially filled out a self-administered questionnaire which included 19 customized open-ended and closed-ended questions based on ICHD (2018) migraine criteria to collect more headache characteristics and place the participants in migraine vs. non-migraine group (Mickleborough et al. 2016).

In the migraine group, each headache attack lasted with a mean score of 12.10 h (SEM = 4.17). The duration of each headache attack in non-migraine headache sufferers had a mean score of 12.23 h (SEM = 5.55). Mean scores of total headache frequency in the past three months were 5.19 (SEM = 1.22) for the migraine group and 2.26 (SEM = 0.41) for the non-migraine headache sufferers. We also looked at headache frequency among those migraineurs who could be potentially categorized into subtypes of migraine with aura and migraine without aura. The mean scores of reported headache frequency were 6.35 in the migraineurs with aura (SEM = 2.19) and 4.12 (SEM = 0.98) in migraineurs without aura. More information on participants’ headache characteristics in migraine and control non-migraine headache groups is found in Tables 1 and 2.

**Table 1:** The main headache characteristics in the migraine and non-migraine groups

	Side *N* (%)		Pain *N* (%)		Intensity *N* (%)			Exercise *N* (%)			Functionality *N* (%)			Nausea *N* (%)	
	One side	Both sides	Pressure	Pulsating	Mild	Moderate	Severe	Has no effect	Makes it worse	Makes it better	Impaired	Normal	Debilitating	Nausea or vomiting	No nausea or vomiting
Migraine	27 (67.5%)	13 (32.5%)	20 (50%)	20 (50%)	4 (10%)	24 (60%)	12 (30%)	3 (7.5%)	36 (92.3%)	0	19 (48.7%)	3 (7.7%)	17 (43.6%)	21 (52%)	19 (47.5%)
Non-migraine	9 (30%)	21 (70%)	19 (65.5%)	10 (34.5%)	19 (63.3%)	11 (36.7%)	0	1 (3.2%)	3 (9.7%)	27 (87.1%)	5 (16.1%)	9 (29.0%)	17 (54%)	1 (3.2%)	30 (96.8%)

**Table 2:** The reported visual symptoms in migraine and non-migraine groups (e.g., flickering lights in vision, spots or lines in vision, loss of vision, sensitivity to light)

Visual symptoms	No visual symptoms *N* (%)	1–2 visual symptoms *N* (%)	≥ 3 visual symptoms *N* (%)
Migraine	11 (27.5%)	23 (57.5%)	8 (15%)
Non-migraine	23 (69.6%)	8 (24.2%)	2 (6.06%)

### EEG recording apparatus

**Fig. 1:**
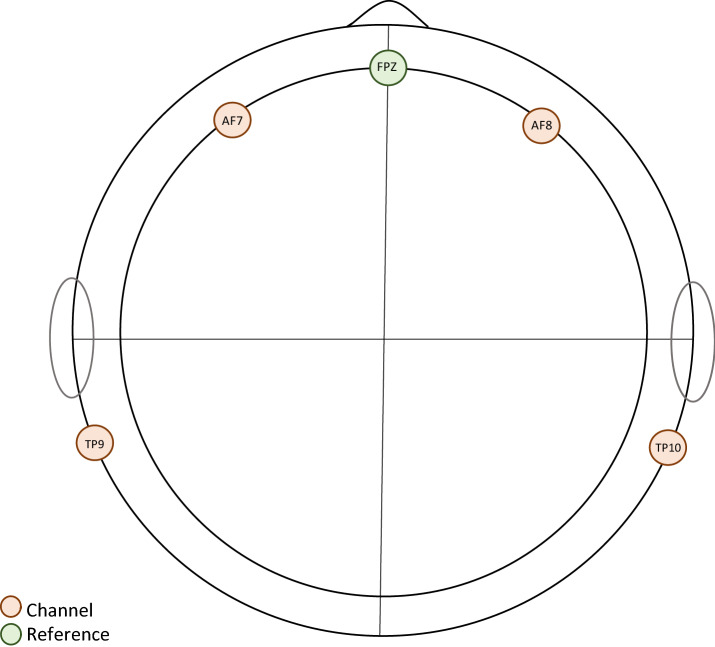
The position of the MUSE electrodes on scalp. AF7, AF8, TP9 and TP10 are channel electrodes and FPZ is marked as the reference electrode in this device. In this study, the data is pooled from AF7 and AF8 for analysis

This study used a portable EEG headband called MUSE (Interaxon, Ontario, Canada) with 500 Hz sampling rate and no onboard data processing. The MUSE EEG system comprises 4 electrodes (AF7, AF8, TP9, and TP10) analogous to electrode Fpz which is considered the reference electrode (Fig. 1). The device is light-weighted and does not require gel application; however, the skin of the forehead and mastoids were dampened to enhance electrical conductance. Like a standard non-portable EEG, the MUSE headband is sensitive to motor movements; therefore, the participants were asked to remain still throughout the whole process of data collection. The EEG data were processed in MATLAB_R2020a based on the protocol earlier utilized by Krigolson et al. (2017) using Brain Vision Analyzer software. The final data were then transferred to SPSS 27.0 for data analysis.

### The oddball task procedure

**Fig. 2:**
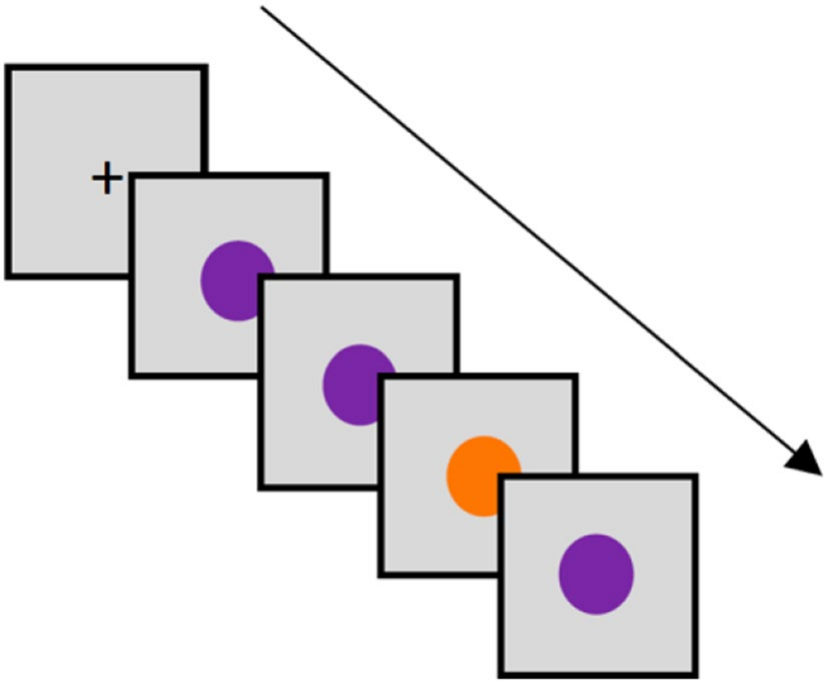
The oddball paradigm test: Each block of trials included 75% of the standard stimulus (purple) for 75% and 25% of the oddball stimulus (orange). The stimuli were presented at random intervals at each block after the fixation cross. Each stimulus lasted for 800–1200 ms at the center of the dark gray screen

The oddball paradigm task was performed in our lab’s soundproof booth on an iPad mini (Apple Inc., California, USA). After completing the self-administered questionnaire, the participants were instructed by a researcher on how to perform the task. They were asked to remain still while being alone in the booth. All the participants were given an identical oddball task while wearing a MUSE EEG Headband. A black fixation cross appeared in the center of the screen (RGB value = [0, 0, 0]) and participants were directed to limit eye movements away from the center of the screen. The participants could see a series of random orange (RGB value = [0 0 255]) and purple (RGB value = [0 255 0]) coloured circles that were set to replace the fixation cross at random intervals and each last for 800–1200 ms in the center of the dark gray screen (RGB value = [108 108 108]). The standard stimulus (orange circle) occurred 75% of the time and comparatively, the target oddball stimulus (the purple circle) was less frequent with 25% of the appearances on the screen (Fig. 2). The circles were presented in random sequence order. Participants were prompted to tap the left side of the screen when viewing the purple stimuli (oddball stimulus) and to tap the right side of the screen when the circle was orange (standard stimulus). All stimuli were presented in front of a light grey background to prevent attentional distraction or discomfort on the participant’s eyes. Stimuli colours and circles were all with the same brightness and dimensions. We used 10 blocks of 50 trials which on average lasted 15 min for each participant to complete. All data were recorded for each session of the study and evaluated on a time-continuum scale.

### ERP analysis

We used MATLAB for raw data analysis and processing in this study (Krigolson et al. 2017). The data was collected from four electrodes AF7, AF8, TP9 and TP10. The data were pooled from AF7 & AF8 electrodes for analysis. The analysis began with data filtering followed by extracting epochs of data from continuous EEG data individually (for the oddball and the control) from 200 ms prior to and 600 ms after stimulus onset. Baseline correction occurred based on the 200 ms before stimulus onset. We subsequently used an artifact rejection algorithm to discard any segment with an absolute difference of more than 60 μv. The remaining segments were averaged for the conditional oddball and standard trials for each participant, then added up to reach grand conditional waveforms (oddball and standard) for migraine and control groups separately. Subsequently, we calculated the grand difference waveform by subtracting the average standard from the average oddball waveforms. The N200 peak component latency was defined (N200: 270 ms) and a time window was picked for calculating the mean peak amplitude of P300 with regard to its double-peaked shape (P300: 330–408 ms). For this means, the voltage amplitudes and latencies at the location between the two peaks of P300 were mean averaged.

## Results

This study used SPSS 27 for statistical analysis. To reassure the validation of our collected ERP data, we conducted a Factorial ANOVA to examine the main effects of ERP conditions (standard vs. oddball) and the groups (migraineurs vs. controls). By looking at the within-subject effects, we found a significant main effect of conditions on the collected ERP responses across the two migraine and control groups [*F* (1,816) = 5.097, *p* = 0.024].

**Fig. 3:**
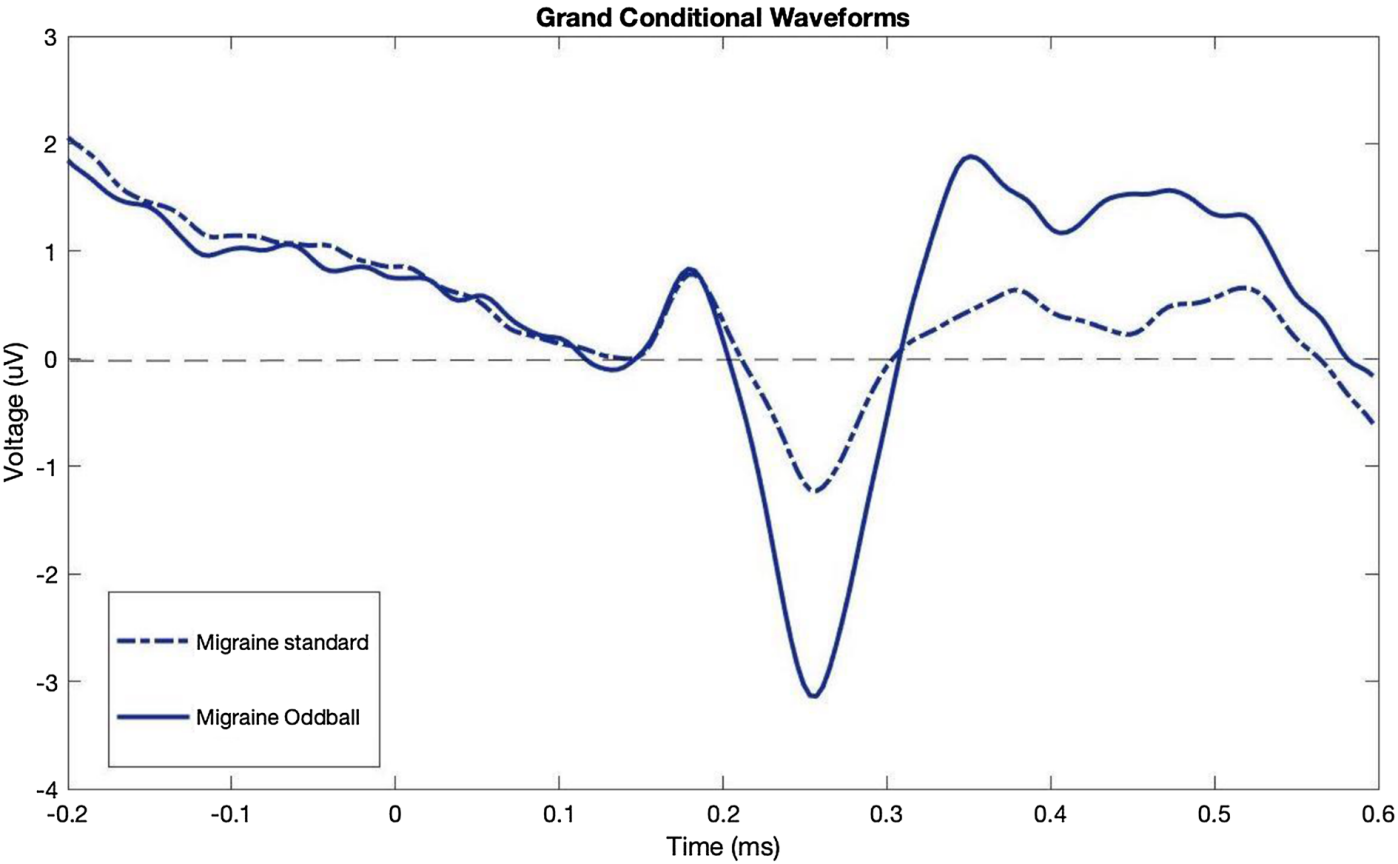
The individual ERP waveforms for Standard and Oddball conditions in migraine

**Fig. 4:**
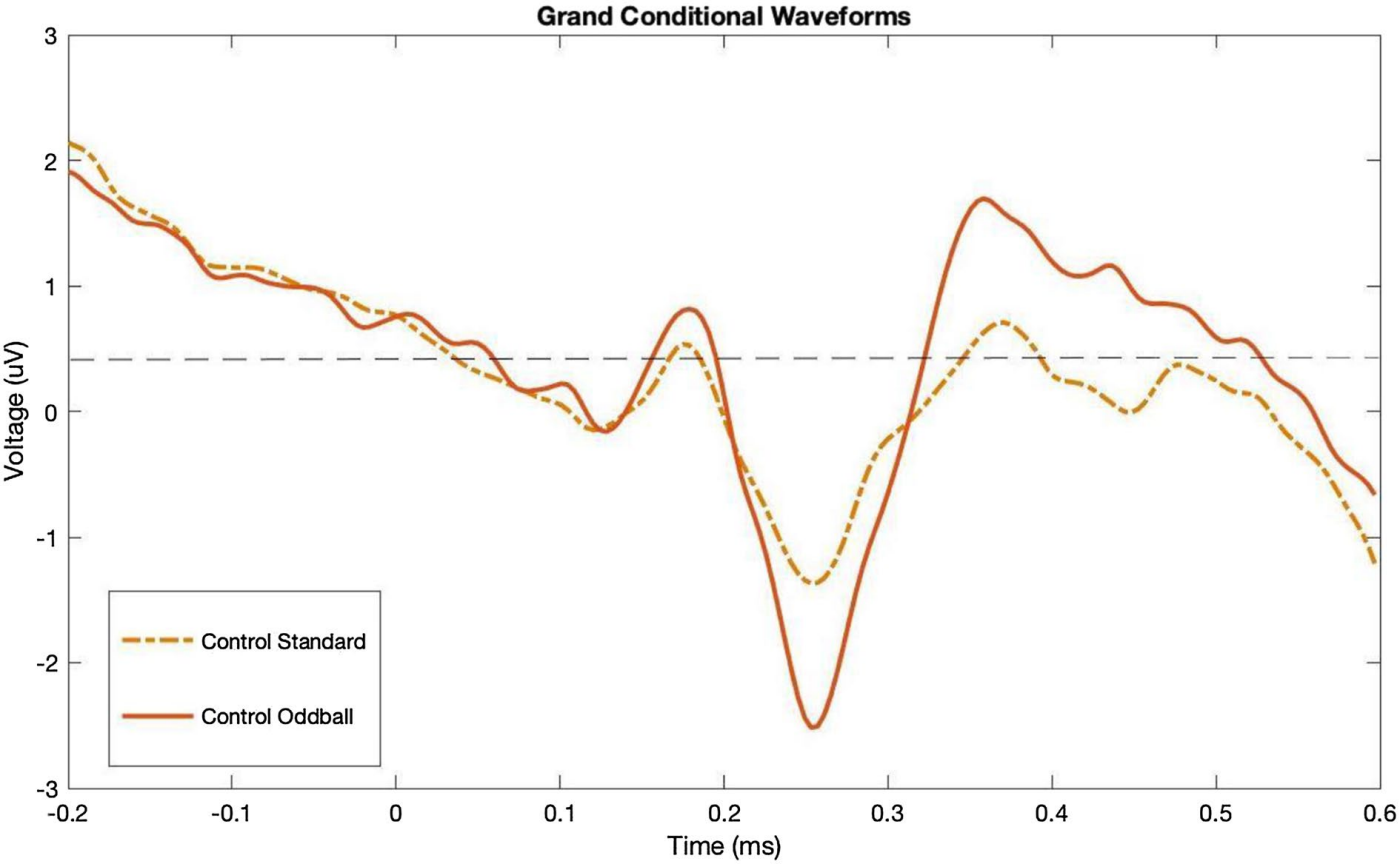
The individual ERP waveforms for Standard and Oddball conditions in migraine

**Fig. 5:**
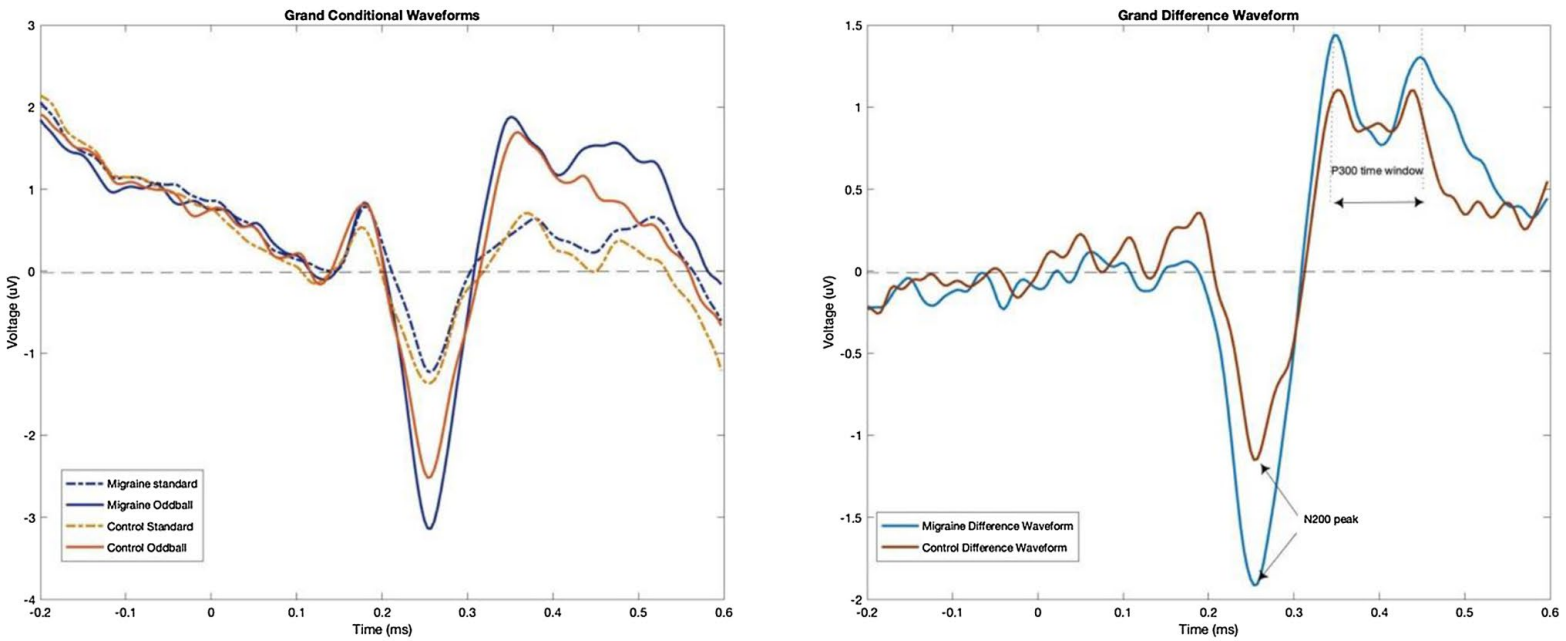
Comparison of the Grand Difference waveforms in migraine and control groups

For N200 component, we looked at the second minimum peak that was found on the grand difference waveform where N200 is usually expected. As explained earlier, we extracted the mean peak amplitude by averaging the voltages surrounding the two emerged peaks of P300 by choosing a time window between the two emerged peaks on the grand ERP difference waveform. As can be seen in  Figs. 3 & 4, while the standard waveforms were similar for both groups, the oddball condition waveform was greater in amplitude for the migraine group compared to the control group. In other words, while both groups showed the typical oddball response, the migraine group showed an even larger difference between oddball to standard, reflecting an amplified oddball effect in N200 and P300. Figure 5 shows the N200 peak and P300 time window grand average difference waveforms in the two groups; for both the N200 and P300 amplitudes, the difference scores between oddball vs. standard were statistically greater in migraine participants when compared to non-migraine controls [(*t* (74) = − 2.406, *p* = 0.019); [*t*(74) = 2.169, *p* = 0.033], respectively].

We tried to look at the possibility of associations between the two ERP components and some headache characteristics. No relation was found between headache intensity and N200 amplitude [*r* (73) = − 0.16, *p* = 0.18] or P300 amplitude [*r* (73) = 0.119, *p* = 0.32]. We also didn’t find a significant relation between headache frequency with the amplitudes of N200 [*r* (73) = − 0.02, *p* = 0.887] and P300 [*r* (73) = 0.2, *p* = 0.17]. No sex differences were observed in the N200 amplitude [*t*(75, 21.92) = 0.286, *p* = 0.778] and P300 amplitude [*t*(75, 24.133) = − 0.14, *p* = 0.889] respectively. In addition, there was no significant difference in N200 and P300 *latencies* between the two groups (Tables 3, 4).

**Table 3:** Descriptive statistics of the P300 and N200 peak amplitude and latency for migraine and non-migraine groups in the grand difference waveform

	Groups	*N*	Mean	Std. Dev	Std. Error
P300 amplitude	Migraine	42	2.9576	1.48533	0.22919
	Non-migraine	33	2.2923	1.06809	0.18593
N200 amplitude	Migraine	42	− 2.4427	1.16436	0.17966
	Non-migraine	33	− 1.8253	1.01941	0.17746
P300 latency	Migraine	42	0.4140	0.05414	0.00835
	Non-migraine	33	0.4011	0.05300	0.00923
N200 latency	Migraine	42	0.2740	0.02732	0.00422
	Non-migraine	33	0.2657	0.03097	0.00539

**Table 4:** *T*-test results for comparing the P300 and N200 amplitude and latency of the grand difference waveform for the migraine vs. non-migraine groups

	Levene’s Test for Equality of Variances		*t*	df	Sig	Mean Differences	Std. Error Difference	95% Confidence Interval of the difference	
	*F*	Sig						Lower	Upper
P300 Amplitude	3.086	0.083	2.169	74	0.033*	0.665252	0.30678	− 0.05384	1.27665
N200 Amplitude	1.523	0.221	− 2.406	74	0.019*	− 0.61732	0.25662	− 1.12876	− 0.10588
P300 Latency	0.051	0.822	1.037	74	0.303	0.01294	0.01248	− 0.01193	0.03781
N200 Latency	0.636	0.428	1.229	74	0.223	0.00829	0.00674	− 0.00515	0.02172

For further exploration of the findings, we aimed to discover whether N200 and P300 amplitudes of the grand average difference waveform differed between migraineurs with and without aura (Table 5) compared to the non-migraine controls. The mean amplitude of N200 and P300 were subsequently compared between the three groups by running a One-Way ANOVA (Table 6). As shown in Table 6, the mean score differences were statistically significant for both N200 amplitude (*F*_2,72_ = 3.180, *p* = 0.048) and P300 amplitude (*F*_2,72_ = 3.168, *p* = 0.048). Finally, the Fisher LSD Test of variance was chosen as the post-hoc test in this study to discover which groups’ mean scores showed significant differences for N200 and P300 amplitudes. While the mean difference score of N200 amplitude between migraineurs with aura (*N* = 29, Mean = − 2.43, SD = 0.23) and those of the control group (*N* = 33 *M* = − 1.85, SD = 0.18) was near to the significance level (Mean difference = − 0.58, *p* = 0.052), migraineurs without aura (*N* = 13, *M* = − 2.69, SD.30) had a larger N200 amplitude when compared to the control group (Mean difference = − 0.84, *p* = 0.032). Additionally, P300 amplitude was significantly different between migraineurs without aura (*N* = 13, *M* = 3.43, SD = 0.40) and the control group (*N* = 33, *M* = 2.32, SD = 0.19) (mean difference = 1.11, *p* = 0.016) (Table 7). The EEG datasets analyzed during the current study are available from the corresponding author on reasonable request.

**Table 5:** Descriptive statistics of the P300 and N200 peak amplitude for subgroups of migraine (migraine with aura and without aura) in the grand difference waveform

	*N*	N200 amplitude					P300 amplitude				
		min	max	mean	Std. Error	Std. Dev	min	max	mean	Std. Error	Std. Dev
Migraine with aura	29	− 5.71	− 0.50	− 2.43	0.23	1.25	0.64	6.83	2.77	0.28	1.51
Migraine without aura	13	− 4.63	− 0.90	− 0.269	0.30	1.06	1.79	6.19	3.43	0.43	1.39

**Table 6:** Results of One-Way Analysis of Variance based on visual symptoms between three groups of migraine with aura, migraine without aura and non-migraine controls

Effect		Univariate tests of significance			*F*	Sig
		Sum of squares	*df*	Mean square		
N200 Amplitude	Between Groups	8.212	2	4.106	3.180	0.048*
	Within Groups	89.095	72	1.291		
	Total	97.307	74			
P300 Amplitude	Between Groups	11.143	2	5.571	3.168	0.048*
	Within Groups	121.342	72	1.759		
	Total	132.485	74

**Table 7:** ANOVA Post Hoc: Fisher LSD test of variance

Dependent variable	(I) Group	(J) Group	Mean Difference (I-J)	Std. Error	Sig	(95%) Confidence interval	
						Lower band	Upper band
N200 amplitude	Migraine with aura	Migraine without aura	0.26289	0.39004	0.503	− 0.5152	1.0410
		Non-migraine control	− 0.58029	0.29356	0.052	− 1.1659	0.0054
	Migraine without aura	Non-migraine control	− 0.84318*	0.38634	0.032*	− 1.6139	− 0.0725
P300 amplitude	Migraine with aura	Migraine without aura	− 0.66359	0.45518	0.149	− 1.5716	− 0.2445
		Non-migraine control	0.45346	0.34259	0.190	− 0.2300	1.1369
	Migraine without aura	Non-migraine control	1.11705*	0.45086	0.016*	− 2.0165	− 0.2176

The asterisk shows a meaningful level of significance

## Discussion

Previous research suggests that migraineurs have an amplified attentional response to visual stimuli (Coppola et al. 2009; Mickleborough et al. 2014), and neuroimaging research suggests this may be due to the migraine brain not adequately assessing the relevance of behavioral stimuli leading to an unwanted increased processing of stimuli (Mickleborough et al. 2016). For example, migraineurs show an increase in ERP amplitude (late positive potential) to repeatedly presented visual stimuli across trial blocks while controls showed no significant effect of the block (Mickleborough et al. 2013). Given this, we hypothesized that migraineurs would show an amplified response to the oddball visual stimuli in a visual oddball task. In other words, we expected the migraine cortical activity would show a greater increase in attentional response to an oddball as measured via ERP N200 and P300 components as compared to controls. As hypothesized, both migraineurs and control participants demonstrated the expected relationship with the oddball having a larger amplitude than the standard (Figs. 3 & 4), with migraineurs showing an amplification of these N200 and P300 difference scores (Fig. 2), suggesting migraineurs have an exaggerated attentional response to the oddball stimuli. This provides supporting evidence to the theory that migraineurs could find it more difficult not to attend to unwanted amplified processing of visual stimuli. This could directly affect the top-down attentional processing in regard to inhibition of irrelevant visual stimuli.

Our findings align with previous research that has indicated reduced sensory habituation, attentional deficits, and increased cortical excitability between headache periods in migraineurs (Mickleborough et al. 2011, 2014; Coppola et al. 2009; Siniatchkin et al. 2003). Below we discuss what the N200 and P300 represent in oddball paradigms and what this might mean for migraineurs. 

### N200 amplitude as indication of an exaggerated attentional shift in migraineurs

The N200 component indicates perceptual processes by showing how a subject shifts attention to a visual stimulus (Papaliagkas et al. 2008; Piras et al. 2003; Kappenman et al. 2021). In addition, based on Hoffman (1990), N200 is a stimulus-oriented component that refers to how individuals distinguish the stimulus before giving a motor response. Accordingly, our results suggest that our sample migraineurs detected and shifted attention to the oddball stimuli more excessively than the controls. Fong et al. (2020) suggested that the increase in N200 could reflect that impairment of inhibitory control over the cortical pyramidal cells resulting in a wide neural activity in the visual cortex during visual stimulation (Fong et al. 2020; Sand et al. 2009). This fits with the proposed abnormality in GABAergic inhibitory interneurons in migraineurs (Chronicle et al. 1994). Our results are consistent with those of de Koning et al. (2001), who also found that in the interictal phase, N200 amplitude was heightened for migraine without aura. In previous research, specific high-frequency patterns such as gratings and checkerboard stimuli resulted in a higher spike of N200 in migraineurs (Oelkers et al. 1999); our findings showed that even a visually simple oddball paradigm could cause a distinguished increase in N200 amplitude. Therefore, this component could be considered as a potential indicator of anomalies in migraineurs’ visual hyperexcitability regardless of the choice of stimulus. We imply that migraineurs could find it more challenging to disregard even the simplest irrelevant information in the given input; such an involuntary attentional shift to the unwanted stimuli could further be discussed in relation to migraineurs’ pain intensity and avoidance behaviour during and between migraine attacks. The failure of attentional control could result in an overload of irrelevant and unnecessary data to process may be presumed in close association with the gradual wear and tear of the brain (also described as allostatic load) which is hypothesized to progressively cause migraine chronification.

### P300 amplitude indicates intensified active attentional processing in migraineurs

As explained earlier, P300 could be considered as the most important component in the post-sensory (i.e., cognitive) level of the ERP when individuals are given visual or auditory stimuli. The P300 is reliably reflective of active attentional processing, a term that refers to the state of identifying, encoding, and categorizing a stimulus during an oddball paradigm task (Krigolson et al. 2017). By measuring the grand average difference waveforms in migraineurs compared to the control counterparts, we found an evident heightened migraine-related amplitude in P300, indicating intensified active attentional processing of the oddball stimuli. Compared to N200, P300 has been more investigated in migraine studies and stands as a vivid representation of selective attention and information processing. Previous research suggests that migraineurs have a decrease in attenuation of unattended external (environmental) stimuli when attention is located in another region of visual space (covert visuospatial orienting) (Mickleborough et al. 2016) but migraineurs do show normal attenuation of visual stimuli when attention is directed to an internal train of thought (mind wandering). Kam et al. (2015) used a sustained attention to response task (SART) for migraine and non-migraine individuals, asking them to stay attended to or unattended to some selected visual tasks. This study showed that the P300 was attenuated in migraineurs respective to their non-migraine counterparts when given SART. Our detected increase in P300 amplitude is a evidence of alterations in cognitive processing of external visual stimuli. This supports the subjective complaints that migrainuers have about feeling overwhelmed by most visual stimuli (such as when presented with flashing lights, or a specific checkerboard or striped patterns). We showed that P300 latency was the same for migraineurs and non-migraineurs. Some studies reported a prolonged P300 in the migraine population compared to healthy subject (Huang et al. 2017), indicating that cognitive performance could be delayed in migrainuers. Yet our result did not show a difference in P300 timing when migraneurs were compared to non-migraine headache sufferers. Similar to the findings of Titlic et al. (2015), we speculate that migraineurs spend a similar amount of time on stimulus evaluation as non-migraineur headache sufferers.

### Migraine with vs. without aura

Looking at the differences between the headache categories, we found that although the N200 amplitude difference between migraineurs with aura and non-migraineurs nearly reached significance (*p* < 0.052), the migraineurs without aura showed a significant reduction in both N200 and P300 amplitudes when compared to the non-migraine controls. Regarding such discrepancy between our study’s migraine categories (with and without aura), we would like to bring attention to paucisymtomatic or “symptom-free” auras, a pathophysiological experience that could impact the cortical mechanisms in migraineurs without a symptomatic emergence (Hajdikhani and Vincent 2019) and how they are associated with cortical hyperexcitability. Some migraineurs do not report any evident aura-related symptoms, so their situation could be translated as migraine without aura while their cortical activities may indicate that they are suffering in the same way as migraineurs with aura. A recent study by Hadjikhani and Vincent (2019) indicated that some silent neurological symptoms could specifically occur in the frontal lobe even in the absence of apparent symptomatic lesions, indicating the potential for “silent auras” and suggesting these two groups may not differ physiologically. Although we propose that the discovered neurophysiological responses are a potential means to track migraine irrespective of the reported visual symptomology, there is a need to probe neurophysiological characteristics of migraine, such as intercellular inhibitory processes, for better detection and classification of migraine anomalies. Future investigations could help us learn whether or not all migraine headaches are involved with intrinsic cortical dysfunctions with similar phenomena to cortical spreading depression (CSD), a gradual neuronal and glial depolarization wave that is more frequently found to happen in migraineurs with aura but is recently being hypothesized as migraine pathogenesis (Harriott et al. 2019). In accordance with the attention that International Classifications of Headache Disorders (ICHD) brought to further neuroscientific and clinical studies of migraine without aura (Headache Classification Committee of the International Headache Society 2018), we encourage explorations of glial and intracortical hyperactivity found in prefrontal areas compared to the extrastriate visual cortex activities and how they could be associated with the frequency of reported visual phenomena such as scotoma. In addition to that, future studies could explore in more detail how attentional processing of migraine as a predominant primary headache disorder is similar to and different from secondary headache due to neurological diseases or post-concussion/mTBI, which may also have reported impaired visual disturbances.

Finally, while we used a portable EEG device that could give convenient output about attentional processing in everyday settings, we decided to keep our environment homogeneously controlled for all the participants during this study. Our oddball paradigm was run in a soundproof booth with controlled visual or auditory distractors. Additionally, compared to the conventional 10–20 EEG system, our utilized apparatus is easy to wear and does not cause any discomfort for headache sufferers presumably even if diagnosed with allodynia. We accordingly suggest future studies to explore attentional processing in everyday life contexts in individuals with headaches, including migraineurs and extending to individuals with a secondary headache from concussions, TBIs, and other neurological disorders. This could shed an eye on anticipations of modifying urban light pollution for those who suffer from light sensitivity, specifically migraineurs who are dealing with an ongoing cause of disability and may need lifelong support and adjustments in their living conditions. Along with the neurophysiological measurement of attentional processing, neuropsychological evaluations of executive functions could be added in order to learn migraineurs’ behavioural responses as well.

### Limitations of the study

In our study, participants were instructed to hold the iPad in a comfortable manner so as to limit the movement of the head during the experiment. However, this may present a confound as the iPad—and therefore, the presented stimuli—was not at a homogenous distance from the participants’ face, both within and between subjects. Therefore, data relevant to small muscle and eye movements were not able to be appropriately collected and analyzed. This may have negatively impacted the study’s results, as some effects may be a result of neglecting eye movements, which have an impact on resulting ERP data. Furthermore, we conducted our research in a laboratory setting using university students, which may be taken into consideration for generalizability. Finally, more information could have been gathered regarding participants’ depression or anxiety and a more diverse sample with different genders, age groups, neurological conditions could have been collected, along with demographic information such as education, race/ethnicity, socio-economic status. Moreover, no information was collected about the frequency of migraine vs. non-migraine attacks in the migraine group.

## Conclusion

We explored visual attention processing of migraineurs compared to non-migraine headache sufferers by focusing on N200 and P300 components of ERP data in response to the oddball paradigm—a method for assessing sensory and post-sensory processes following the onset of a stimulus. The migraineurs presented an amplified difference in their post-sensory processing of the visual oddball stimuli in N200 and P300 as compared to controls, indicating that migraineurs detected, shifted attention to, and actively attended the oddball stimuli more excessively than the controls. Overall, we provided further evidence for a heightened cortical response when attending to visual stimuli in migraineurs. Our results imply that migraineurs go through a more complicated attentional processing in response to what they see around them every day, during the headache-free phases of their lives. This study adds to the growing picture that abnormal attentional processing may contribute to an increased brain response to ordinary visual stimuli, and the extent to which this response is related to hyperexcitability of the cortex and headache pain in migraine, as well as how it may relate to other neurological conditions which have similar headache pain and visual sensitivity, such as post-concussion system, warrant future work.
